# Aneurysmal subarachnoid hemorrhage: intensive care for improving neurological outcome

**DOI:** 10.1186/s40560-018-0297-5

**Published:** 2018-05-08

**Authors:** Tomoya Okazaki, Yasuhiro Kuroda

**Affiliations:** 1 Emergency Medical Center, Kagawa University Hospital, 1750-1 Ikenobe, Miki, Kita, Kagawa 761-0793 Japan; 2 Department of Emergency, Disaster, and Critical Care Medicine, Faculty of Medicine, Kagawa University, 1750-1, Ikenobe, Miki, Kita, Kagawa 761-0793 Japan

**Keywords:** Aneurysmal subarachnoid hemorrhage, Delayed cerebral ischemia, Early brain injury, Sympathetic activity, Hemodynamic management, Fever management, Glycemic management, Dysnatremia, Duration of intensive care management

## Abstract

**Background:**

Aneurysmal subarachnoid hemorrhage is a life-threatening disease requiring neurocritical care. Delayed cerebral ischemia is a well-known complication that contributes to unfavorable neurological outcomes. Cerebral vasospasm has been thought to be the main cause of delayed cerebral ischemia, and although several studies were able to decrease cerebral vasospasm, none showed improved neurological outcomes. Our target is not cerebral vasospasm but improving neurological outcomes. The purpose of this review is to discuss what intensivists should know and can do to improve clinical outcomes in subarachnoid hemorrhage patients.

**Main body of the abstract:**

Delayed cerebral ischemia is thought to be due to not only vasospasm but also multifactorial mechanisms. Additionally, the concept of early brain injury, which occurs within the first 72 h after the hemorrhage, has become an important concern. Increasing sympathetic activity after the hemorrhage is associated with cardiopulmonary complications and poor outcomes. Serum lactate measurement may be a valuable marker reflecting the severity of sympathetic activity. The transpulmonary thermodilution method will bring about an advanced understanding of hemodynamic management. Fever is a well-recognized symptom and targeted temperature management is an anticipated intervention. To avoid hyperglycemia and hypoglycemia, performing moderate glucose control and minimizing glucose variability are important concepts in glycemic management, but the optimal target range remains unknown. Dysnatremia seems to be associated with negative outcomes. It is not clear yet that maintaining normonatremia actively improves neurological outcomes. Optimal duration of intensive care management has not been determined.

**Short conclusion:**

Although we have an advanced understanding of the pathophysiology and clinical characteristics of subarachnoid hemorrhage, there are many controversies in the intensive care unit management of subarachnoid hemorrhage. With an awareness of not only delayed cerebral ischemia but also early brain injury, more attention should be given to various aspects to improve neurological outcomes.

## Background

Aneurysmal subarachnoid hemorrhage (SAH) is known to be associated with high mortality, morbidity, and burden of healthcare [1, 2]. SAH is one of the main targets of neurocritical care [3–5]. Delayed cerebral ischemia (DCI) is a well-known complication that usually develops in one third of SAH patients between 4 and 14 days after the hemorrhage [2]. A definition of DCI for clinical trials and observational studies was proposed in 2010 [6]. Cerebral vasospasm was thought to be the main cause of DCI, and several studies on the prevention of cerebral vasospasm have been conducted. As an example, clazosentan, an endothelin receptor antagonist, significantly decreased vasospasm compared with placebo [7] but failed to improve functional outcome [8]. A randomized controlled trial (RCT) of fasudil reached the same results [9]. These findings suggested two possibilities: First, factors other than cerebral vasospasm have an important role in the development of DCI. Second, factors other than DCI have profound effects on neurological outcomes. Our target is not cerebral vasospasm but improving neurological outcomes. The purpose of this review is to discuss what intensivists should know and can do to improve clinical outcomes in SAH patients.

## Review

### Concept of DCI and early brain injury

Although a clear picture of DCI remains unknown, human and animal studies have suggested that several pathophysiological mechanisms contribute to development of DCI. These mechanisms are cerebral vascular dysregulation, including cerebral vasospasm and microcirculatory dysfunction, microthrombosis, cortical spreading depolarization, and neuroinflammation [10]. We cannot discuss each factor in detail in the current review.

A concept of early brain injury (EBI) for the immediate brain injury during the first 72 h after the hemorrhage has been proposed over the past years [11]. EBI is another factor that affects neurological outcome. Aneurysmal rupture leads to transient global ischemia, which is caused by increasing intracranial pressure, decreasing cerebral perfusion pressure, and decreasing cerebral blood flow, and toxic activity of the subarachnoid hemorrhage. These mechanisms induce multifactorial derangement, such as microcirculatory constriction, endothelial cell apoptosis, blood–brain barrier disruption, brain edema, and thromboinflammatory cascade [1, 12].

**Fig. 1:**
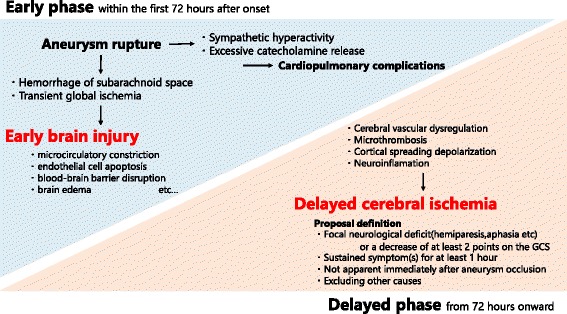
Overview of early brain injury and delayed cerebral ischemia in subarachnoid hemorrhage patients

Figure 1 shows an overview of EBI and DCI. It is important to bear in mind that both DCI and EBI involve multiple pathophysiological factors. It helps us to interpret basic and clinical trials in the past and future.

### Management of sympathetic activity

Increasing sympathetic activity and excessive catecholamine release have received constant attention [13]. Recent studies have shown that acute catecholamine surge occurring immediately after the hemorrhage resulted in cardiopulmonary complications such as neurogenic-stunned myocardium and neurogenic pulmonary edema [14]. An association between serum catecholamine concentration and poor neurological outcome has been reported in some observational studies [13, 15].

Serum lactate measurement is very easy and common in intensive care units (ICUs). Elevated serum lactate level is due to not only tissue hypoxia but also aerobic glycolysis caused by excessive catecholamine release [16]. Elevated serum lactate levels during the acute phase in SAH patients seem natural in theory. A retrospective study with 145 patients revealed the alteration of serum lactate levels during ICU stays [17]. In this study, the elevated serum lactate levels on admission gradually decreased to the normal range. Three retrospective observational studies found that elevated serum lactate levels in the acute phase were associated with mortality and poor neurological outcomes [17–19]. Although further studies are warranted, serum lactate measurement may be a valuable marker reflecting the severity of sympathetic activity and excessive catecholamine release. However, serum lactate level is significantly affected by anaerobic glycolysis. Physicians should identify factors causing decreased oxygen delivery to tissues that may result from conditions such as cardiogenic or hypovolemic shock, sepsis, and severe anemia [20]. As discussed in the later sections, fluid volume status is crucial in the ICU management of SAH patients. To the best of our knowledge, there are no reports regarding the utility of lactate-guided evaluation of volume status in SAH patients.

There have been few studies on the management of sympathetic activity. A meta-analysis of three retrospective studies showed that preadmission beta blockers did not decrease cardiac dysfunction or mortality [21]. The association of dexmedetomidine with neurological outcomes was explored in a single-center retrospective observational study [22]. This study found that low-dosage dexmedetomidine during the first 24 h after admission had better lactate clearance and was associated with favorable neurological outcomes. However, there were many confounding factors in this study, and the causal relationship between dexmedetomidine and clinical outcomes remains unknown.

### Hemodynamic management

Although prophylactic triple-H therapy (hypervolemia, hypertension, hemodilution) for preventing DCI was acceptable [23], current evidence does not support its efficacy and recommends maintaining normovolemia [24–26]. Induced hypertension and volume status have been regarded as important.

Based on several case series, induced hypertension was a highly anticipated intervention for patients with DCI [27]. An RCT was designed to evaluate the effectiveness of induced hypertension; however, this trial was prematurely terminated because of its ineffectiveness for cerebral perfusion and slow recruitment [27]. Currently, there is no way to treat DCI definitively. Therefore, we should perform appropriate interventions, including induced hypertension and endovascular treatment, according to the needs of individual patients [28].

It is difficult to accurately evaluate volume status and maintain normovolemia. The transpulmonary thermodilution (TPTD) method can measure various hemodynamic parameters [29]. Several studies have reported the utility of TPTD in SAH management. A multicenter prospective cohort study showed that a lower global end-diastolic volume index as an indicator of cardiac preload during the first week was associated with the occurrence of DCI, and its threshold was slightly higher than the normal value (822 mL/m^2^) [30]. Additionally, the association between prophylactic triple-H therapy and global end-diastolic volume index was evaluated using the TPTD study data [31]. Accordingly, patients were divided into two groups based on whether or not they were under prophylactic triple-H therapy. Patients in the triple-H therapy group had a greater amount of fluid than the other group, but there were no significant differences in the global end-diastolic volume index and clinical outcomes between the groups. A physician-driven triple-H therapy could not effectively increase the global end-diastolic volume index; therefore, previous studies on triple-H therapy may fail to improve clinical outcomes. An RCT was performed to assess the efficacy of TPTD-based management compared with fluid balance or central venous pressure-guided management [32]. However, TPTD-based management neither decreased DCI nor improved functional outcomes. A possible reason is that the TPTD-based management protocol was not established using an SAH-specific cutoff value. Although there will be additional knowledge concerning hemodynamics in SAH gained from the use of the TPTD method, how to translate the TPTD evidence for use in less invasive methods is a future issue.

### Fever management

Fever is defined as a body temperature > 38.3 °C and is a well-recognized symptom experienced by 70% of patients with SAH [33, 34]. Poor clinical grade on admission and intraventricular hemorrhage are regarded as risk factors for fever in SAH patients [34]. Several retrospective or prospective observational studies showed that fever was significantly associated with mortality and poor neurological outcome [34–36]. Based on these findings and those from studies with animal SAH models clarifying the neuroprotective effect of targeted temperature management (TTM) [37, 38], the benefit of TTM for SAH patients was assessed in several studies. Studies exploring the association between TTM and clinical outcomes are summarized in Table 1 [39–43]. A study of TTM for refractory intracranial pressure elevation was excluded [44]. As indicated in Table 1, there are many differences among the studies with regard to the purpose of TTM (treatment of refractory fever or prevention of fever), protocol of TTM (initiation timing, target temperature, duration of target temperature, and rewarming rate), and method of TTM. These differences may produce varying results. A prospective, multi-center RCT to evaluate the efficacy of TTM (32–35 °C for a minimum of 5 days) for patients with poor-grade SAH is registered on ClinicalTrials.gov [45]. Although this trial will not be able to provide solid answers, it will provide some information regarding when, how, and on whom TTM is to be performed.

**Table 1:** Summary of targeted temperature management studies

Article	Design	Patient	Intervention or exposure	Comparison	Main results
Muroi C 2008 [39]	Single-center, prospective cohort study	SAH patients with a ventricular catheter for cerebrospinal fluid drainage	(1) 33 °C with an endovascular cooling device (2) Barbiturate coma *N* = 7	No detail described *N* = 8	There was no significant difference in neurological outcome (GOS > 3) at 1 year (42.9 vs. 50.0%).
Anei R 2010 [40]	Single-center Before–after study	Poor-grade SAH patients (WFNS scale > 3)	(1) Induction within 24 h after the hemorrhage (2) 34 °C for 48 h with an cooling blanket (3) Rewarming at the rate of 1 °C/24 h *N* = 16	No detail described *N* = 19	There was no significant difference in neurological outcome at 3 months.
Badjatia N 2010 [41]	Matched controlled analysis from single-center, prospective cohort	SAH patients with antipyretic-resistant fever	37 °C for 14 days with a surface cooling device *N* = 40	Oral acetaminophen with or without use of a water-circulating blanket *N* = 80	In multivariate analysis, TTM was associated with better neurological outcome at 12 months (79 vs. 54%).
Kuramatsu JB 2015 [42]	Matched controlled analysis from single-center, prospective cohort	Poor-grade SAH patients (Hunt and Hess grade > 3 and WFNS scale > 3)	(1) Induction within 48 h after the hemorrhage (2) 35 °C for 7 days with an endovascular cooling device (3) Rewarming at the rate of 0.5 °C/24 h *N* = 12	Intravenous paracetamol *N* = 24	Patients in TTM groups had a significantly lower incidence of DCI (50.0 vs. 84.5%) and a tendency to have better functional outcome (mRS < 3) at 6 months (66.7 vs. 33.3%).
Choi W 2017 [43]	Single-center, randomized control trial	Poor-grade SAH patients (Hunt and Hess grade > 3 and modified Fisher scale > 2)	(1) Induction as soon as possible after ruptured aneurysmal treatment (2) 34.5 °C for 48 h with an endovascular cooling device or a surface cooling device (3) rewarming at the rate of 1 °C/24 h to 36.5 °C *N* = 11	No detail described *N* = 11	There were no significant differences in the incidences of DCI (36.3 vs. 45.6%) and favorable neurological outcome (mRS < 3) at 3 months between two groups (27.3 vs. 9.1%).

*SAH* subarachnoid hemorrhage, *TTM* targeted temperature management, *WFNS* World Federation of Neurosurgical Society, *DCI* delayed cerebral ischemia, *mRS* modified Ranking scale score, *GOS* Glasgow outcome scale

In the current scenario, clinicians must clearly not neglect efforts to identify potential causes of fever. In case of TTM, controlling shivering should be emphasized.

### Glycemic management

Hyperglycemia is frequently observed in SAH patients and is independently associated with poor outcomes [26]. Although hyperglycemia on admission may be merely a marker of severity, an association of worse outcomes with persistent hyperglycemia has been reported [46], and an early correction of hyperglycemia is considered reasonable. The European Stroke Organization guidelines recommend that hyperglycemia with blood glucose > 10 mmol/L (180 mg/dL) should be treated [26]. However, there is no evidence supporting this cutoff value.

Hypoglycemia is associated with negative outcomes in SAH patients [47] as in general critically ill patients [48]. However, the optimal cutoff value of hypoglycemia in SAH patients has not been established [24–26]. A retrospective observational study found that > 50% of SAH patients with minimum glucose < 8 mmol/L (90 mg/dL) had unfavorable outcomes at discharge [49]. Especially during insulin infusion, cerebral interstitial hypoglycemia has been shown to occur despite the absence of blood hypoglycemia in two cerebral microdialysis studies [50, 51]. It may be innocuous to set a higher threshold in SAH patients than in critically ill patients.

Optimal glycemic control in SAH patients has been discussed in two before–after studies and one RCT (Table 2). One before–after study showed that a strict glucose control regimen (5.0–6.7 mmol/L; 90–120 mg/dL) failed to reduce mortality and was associated with the incidence of hypoglycemia [52]. Another before–after study showed that an aggressive hyperglycemia management protocol (4.4–7.8 mmol/L (80–140 mg/dL)) did not improve time trend-adjusted neurological outcomes [53]. An RCT performed with a small number of patients after surgical clipping found that intensive insulin therapy (4.4–6.7 mmol/L; 80–120 mg/dL) compared with maintaining blood glucose < 11.1 mmol/L (200 mg/dL) significantly reduced infection rates as the primary endpoint, but there was no significant difference in the neurological outcomes between the two groups [54]. On the basis of the above findings, it seems that strict glycemic control provides little benefit and increases the risk of hypoglycemia.

**Table 2:** Summary of glycemic control studies

Article	Design	Patient	Intervention or exposure	Comparison	Outcome	Main results
Thiele RH 2009 [52]	Single-center Before–after study	Patients with the primary diagnosis of SAH	5.0–6.7 mmol/l (90-120 mg/dl) *N* = 491	No detail described *N* = 343	In-hospital mortality	Three was no significant difference in in-hospital mortality.
Latorre JG 2009 [53]	Single-center Before–after study	SAH patients with blood glucose > 11.1 mmol/l (200 mg/dl) on admission or the first 24 h mean blood glucose > 7.8 mmol/l (140 mg/dL)	4.4–7.8 mmol/l (80-140 mg/dl) *N* = 166	≦ 11.1 mmol/l (200 mg/dl) *N* = 166	mRS ≧ 4 at 3–6 months	Given temporal trend, there was no significant difference in neurological outcome.
Bilotta F. 2007 [54]	Single-center, randomized control trial	SAH patients undergoing emergency cerebral aneurysm clipping	4.4–6.7 mmol/l (80–120 mg/dl) *N* = 40	4.4–12.2 mmol/l (80–220 mg/d) *N* = 38	mRS ≧ 4 at 6 months (as secondary outcome)	There was no significant difference in neurological outcome.

*mRS* modified Ranking Scale score

Three retrospective observational studies have suggested that glucose variability was associated with cerebral infarction, mortality, and poor neurological outcomes [49, 55, 56]. A cerebral microdialysis study of 28 comatose SAH patients showed that systemic glucose variability was associated with cerebral metabolic distress [56]. Another microdialysis study found that an acute decrease in blood glucose, despite being within normal range, was associated with brain energy metabolic crisis and an elevated lactate/pyruvate ratio [57]. We should be careful not to make extensive changes in blood glucose concentrations.

In addition to these issues, there are two questions about glycemic management in SAH patients: First, what is the effect of pre-existing impaired glucose tolerance? Previous studies have reported that diabetic status affected the association of hyperglycemia, hypoglycemia, and glucose variability with mortality in critically ill patients [58, 59]. Second, can we apply the same glycemic management during both the EBI and DCI period? These questions have never been explored.

### Dysnatremia management

Both hyponatremia and hypernatremia commonly occur in the ICU management in SAH patients [25]. However, there are few studies describing the characteristics of sodium alteration in ICU. A single-center retrospective observational study showed that serum sodium concentrations increased for the first few days and decreased to the nadir level at 6–12 days [60]. A similar trend was observed in another retrospective study [61].

Hyponatremia, defined as serum sodium levels < 135 mmol/L, occurs in one third of SAH patients [62] and is triggered by multifactorial causes, which include hypovolemia, a syndrome involving inappropriate secretion of antidiuretic hormone, glucocorticoid deficiency, and cerebral salt-wasting syndrome and its interactions [63, 64]. Although a systematic review showed that hyponatremia was associated with longer hospitalization and cerebral infarction, whether hyponatremia affects neurological outcomes remains controversial [62]. The threshold of hyponatremia associated with poor neurological outcomes was addressed in a retrospective observational study involving 131 patients [60]. In this study, multiple regression analysis showed that minimum sodium levels in the ICU were associated with unfavorable neurological outcomes at hospital discharge, and receiver operating characteristics curve analysis derived a cutoff value of 132 mmol/L. According to a systematic review on prevention and treatment of hyponatremia, mineralocorticoids use reduced natriuresis and volume contraction but did not improve neurological outcomes [65].

Hypernatremia is commonly defined as serum sodium levels > 145 mmol/L and develops less frequently than hyponatremia [60]. SAH-related hypothalamic dysfunction induces central diabetes insipidus followed by hypernatremia [66, 67]. The association of hypernatremia with clinical outcomes is summarized in Table 3 [67–72].

**Table 3:** Summary of the association between hypernatremia and clinical outcomes

Article	Study design	Definition of hypernatremia (mmol/L)	Number of patient with hypernatremia/total number of patients	In-hospital mortality	Delayed cerebral ischemia	Neurological outcome
Qureshi AI 2002 [68]	Post hoc analysis of RCT	> 145	58/298 (19.5%)	NA	NA	Worse at 3 months
Wartenberg KE 2006 [69]	Post hoc analysis of single-center, prospective cohort study	> 150	91/576 (15.8%)	NA	NA	Not significant at 3 months
Fisher LA 2006 [70]	Post hoc analysis of single-center, prospective cohort study	> 143	48/214 (22.4%)	Not significant	NA	NA
Beseoglu K 2014 [67]	Single-center, retrospective cohort study	> 145	82/264 (31.1%)	NA	NA	Worse at 12 months
Lantigua H 2015 [71]	Post hoc analysis of single-center, prospective cohort study	> 150	250/1200 (20.8%)	Higher	NA	NA
Spatenkova V 2017 [72]	Single-center, retrospective and prospective observational study	> 150	41/334 (11.9%)	Higher	NA	Worse at 12 months
Okazaki T 2017 [60] ^a^	Single-center, retrospective observational study	≧ 145	40/131 (30.5%)	NA	NA	Worse at hospital discharge

*NA* not applicable

^a^Result from additional data analysis

Referring to this summary, the optimal threshold seemed to be 145 mmol/L, as suggested in a previous study [60], and hypernatremia was associated with poor outcomes. The preventive effect of a specific drug or protocol on hypernatremia has not been tested to date.

Given the above findings, it remains unknown if there is a causal relationship between dysnatremia and clinical outcomes. Additional studies are required to evaluate whether maintaining normonatremia actively improves neurological outcomes.

### Duration of intensive care management

As discussed, physicians should perform multimodality monitoring and optimal intervention as needed for SAH patients. The duration of continuing intensive care, especially in stable patients without neurological deficit after aneurysmal treatment, should be determined. Subgroup analysis in SAH patients with Hunt and Kosnik grades I–II of a single-center before–after study gave us some clues regarding the duration of continuing intensive care [5]. This study showed that neurointensivist-managed ICU implementation was associated with improved neurological outcome and with prolonged ICU stay [median (interquartile range), 12 (9–14.3) vs. 3 (1.5–10.5) days, *p* < 0.01]. Another before–after study exploring the beneficial effect of neurointensivist on discharge disposition also demonstrated similar results (length of ICU stay: mean ± standard deviation days, 11.6 ± 11.0 vs. 3.7 ± 12.4, *p* < 0.01) [4]. Approximately 12 days of ICU stay, as observed in these studies, may roughly indicate the duration required to prevent, detect, and deal with subsequent complications. However, further studies are warranted to determine whether longer ICU stay can contribute to improved outcomes in good-grade SAH patients and whether ICU stay can be safely reduced.

## Conclusions

Although we have an advanced understanding of the pathophysiology and clinical characteristics of SAH, there are many controversies in the ICU management of SAH. With an awareness of not only DCI but also EBI, more attention should be given to various aspects, including sympathetic activity, hemodynamic management, glycemic management, dysnatremia, and duration of intensive care management to improve neurological outcomes.

## Abbreviations

DCI: Delayed cerebral ischemia
EBI: Early brain injury
ICU: Intensive care unit
RCT: Randomized controlled trial
SAH: Subarachnoid hemorrhage
TPTD: Transpulmonary thermodilution
